# Effects of Hypoxic Environment on Periodontal Tissue through the ROS/TXNIP/NLRP3 Inflammasome Pathway

**DOI:** 10.1155/2022/7690960

**Published:** 2022-01-17

**Authors:** Rui Zhu, Xiaohui Mi, Yongming Li

**Affiliations:** Department of Orthodontics, Shanghai Engineering Research Center of Tooth Restoration and Regeneration, School and Hospital of Stomatology, Tongji University, Shanghai, China

## Abstract

There is low evidence for the possible association between obstructive sleep apnea-hypopnea syndrome (OSAHS) and periodontitis, necessitating further research. This study was aimed at investigating this association. For the in vitro study, 8-day-old Wistar rats were divided into the unilateral nasal obstruction group (UNO) and the sham surgery group (SHAM). Rats in the former group were subjected to UNO by cauterization of the external nostril at the age of 8 days. Immunofluorescence analysis, quantitative real-time polymerase chain reaction, and western blot were performed to assess the expression of thioredoxin-interacting protein (TXNIP), NLR family pyrin domain-containing 3 (NLRP3) inflammasome-associated factors, and interleukin-1*β* (IL-1*β*). Throughout the experimental period, the weights of rats in the two groups were similar. The mRNA and protein expression of *TXNIP* and *IL-1β* was significantly higher in the UNO than in the SHAM groups. Compared with SHAM, NLRP3 inflammasome-associated factors were activated in the UNO group. For the in vitro study, a cellular hypoxia model was established by treating human periodontal ligament cells (HPDLCs) with cobalt chloride. The studies showed that hypoxia can induce an excessive production and accumulation of reactive oxygen species (ROS) in HPDLCs and induce abnormal expression of TNXIP, NLRP3 inflammasome-related factors, and IL-1*β*. More importantly, N-acetylcysteine induced reduction of ROS in HPDLCs, downregulated TXNIP expression, inhibited the expression and aggregation of NLRP3 inflammasome-related factors, and abrogated the inflammatory response to hypoxia. In conclusion, hypoxia-induced ROS can activate the TXNIP/NLRP3 inflammasome signaling pathway in response to oxidative stress, resulting in the increased expression of inflammatory factors in HPDLCs. Our findings provide evidence for the mechanism underlying the possible association between OSAHS and periodontal disease.

## 1. Background

Obstructive sleep apnea-hypopnea syndrome (OSAHS) is a condition characterized by obstructive sleep apnea and insufficient ventilation caused by a collapse or blockage of the upper airway during sleep. Sleep defects, daytime naps, fatigue, and frequent drops in oxygen saturation (SpO_2_) are associated with this condition. The incidence of OSAHS in the United States is 2–4% [1]. OSAHS patients are in a hypoxic state and often have closely related complications, such as cardiovascular disease, cognitive decline, and altered cranial and maxillofacial development [2–4]. The incidence of periodontal disease in patients with OSAHS is higher than that in healthy people [5–7].

Periodontal disease is initiated and sustained by the imbalance between the oral microbial community in microbial biofilms and the host inflammatory response [8, 9]. It is characterized by its occurrence in the supporting tissues of the teeth, including gingival disease involving only gingival tissues and periodontitis affecting deep periodontal tissues, thereby causing alveolar bone resorption and ultimately leading to tooth loss [10]. Further, periodontal disease is associated with a variety of systemic diseases, such as cardiovascular diseases, diabetes, and inflammatory bowel disease [11, 12].

The upregulation of inflammatory mediators in periodontal tissue can lead to the occurrence and development of disease. The inflammatory factor IL-1*β* is involved in the pathogenesis of periodontitis and has been widely studied because it stimulates the recruitment and differentiation of osteoclasts in tissues and contributes to bone absorption during periodontitis [13]. Inflammasomes are multiprotein complexes that can induce inflammatory responses in cells and play an important role in the inflammatory response of various tissues. Among them, the nucleotide-binding leucine-rich repeat (NLR) pyrin domain-containing 3 (NLRP3) inflammasome is the most prominent in the NLR family and is proven to be involved in innate immune responses to infection, inflammation, and chronic diseases [14]. The NLRP3 inflammasome complex is composed of NLRP3, recruitment domain (ASC), and caspase-1 [15]. In response to tissue sensing inflammatory stimuli, procaspase-1 is activated and cleaves IL-1*β* into its biologically active form. Previous studies have shown that hypoxia can regulate the NLRP3 inflammasome expression through TXNIP expression [16, 17]. Meanwhile, overexpression of NLRP3 in gingival tissue and increased NLRP3 salivary levels have been observed in patients with periodontitis [18]. The ROS/TXNIP/NLRP3 inflammasome signaling pathway may play an important role in the pathogenesis of periodontal disease. Patients with OSAHS are hypoxic, and the tissues often have oxidative stress. When the body is in a hypoxic state, too many reactive oxygen species (ROS) in the respiratory chain complex cannot be cleared in time, leading to adverse reactions, such as cell DNA damage, resulting in cell dysfunction and even death. This response is known as oxidative stress [19]. Previous studies have shown that upon its separation from thioredoxin, thioredoxin-interacting protein (TXNIP) expression levels increase, a necessary process leading to oxidative stress in the body. Increased TXNIP expression levels can activate NRLP3 inflammasomes directly and eventually induce an inflammatory response [20–22].

Current studies have shown that there appears to be low evidence for the association between OSAHS and periodontitis [23], and the pathophysiological mechanisms underlying the association between the two conditions remain unclear. We hypothesized that there is a possible correlation between OSAHS and periodontal disease. OSAHS may affect the incidence of periodontal diseases through the ROS/TXNIP/NLRP3 inflammasome signaling pathway.

This study was aimed at elucidating and providing evidence for the pathophysiological mechanism underlying the correlation between OSAHS and periodontal diseases.

## 2. Materials and Methods

### 2.1. Isolation and Culture of HPDLCs

Healthy periodontal tissue was collected from five healthy candidates (16–26 years old; mean 17.8 years) who underwent tooth extraction as an orthodontic treatment. The teeth were placed in sterile phosphate-buffered saline (PBS; Hyclone, USA) immediately after extraction. The periodontal ligament tissues were scraped from the middle third of the tooth roots and then digested for 30 min at 37°C in 3 mg/ml collagenase type I (Sigma, USA). The resulting cell suspension was seeded in 25 cm^2^ flasks containing alpha-minimal essential medium (Hyclone, USA) supplemented with 10% fetal bovine serum (GIBCO, USA) and 1% penicillin/streptomycin. The cells were incubated at 37°C in a humidified 5% CO_2_ incubator. Cells between the third and sixth generations were used for the subsequent experiments. This research protocol was approved by the Ethical Committee of the Tongji University College.

### 2.2. Cell Counting Kit-8 Assay (CCK-8)

The CCK-8 assay (Beyotime, China) was used to evaluate the effect of different concentrations of CoCl_2_ on cell proliferation. HPDLCs were seeded in 96-well plates at a density of 2000 cells/well in 100 *μ*l alpha-minimal essential medium, and then, different concentrations (0, 100, 200, 300, and 400 *μ*M) of CoCl_2_ were added to stimulate cells for different periods (24 and 48 h). After 2 h of culture with 10 *μ*l/well CCK-8, the fluorescence intensity was measured at 450 nm. Cell proliferation was plotted relative to the untreated controls.

### 2.3. CoCl_2_ Treatment to Mimic Hypoxic Treatment

CoCl_2_ was dissolved directly in the cell culture medium and filtered by a 0.2 *μ*m filter to produce a concentrated solution of the culture medium. To mimic the hypoxic environment in vivo, the cells were treated with or without CoCl_2_ at varying concentrations (200 and 400 *μ*M) for varying times (24 and 48 h). As part of the experiments, some cells were pretreated with the ROS inhibitor N-acetylcysteine (NAC; 10 mM) for 2 h before CoCl_2_ (200 *μ*M) exposure for 24 h.

### 2.4. Reverse Transcription-Polymerase Chain Reaction (RT-PCR)

Total RNA from rat periodontal tissues and HPDLCs was isolated using a TRIzol® reagent (Takara Ltd., Otsu, Japan), and cDNA was synthesized using PrimeScript RT Master Mix (Takara Ltd.). RT-PCR was conducted using qPCR SYBR Green Master Mix (Yeasen, China) and a LightCycler® 96 instrument (Roche, Germany). Primer sequences used for RT-PCR (Sangon, China) are listed in Table 1.

### 2.5. Western Blotting

Periodontal tissues were washed with PBS and lysed with radioimmunoprecipitation assay buffer (Beyotime, China). The tissue lysates were separated on 10% SDS-PAGE gels and transferred to polyvinylidene fluoride membranes (Millipore, USA). Membranes were blocked with 5% skimmed milk in TBST for 1 h at room temperature and then incubated with the following primary antibodies overnight at 4°C: TXNIP (Rabbit IgG, Boster, China), NLRP3 (Rabbit IgG, Boster, China), ASC (Rabbit IgG, Immunoway, China), and caspase-1 (Rabbit IgG, Immunoway, China) at 1 : 1000. Thereafter, they were incubated with antirabbit IgG secondary antibody (CST, USA) at 1 : 5000 at room temperature for 2 h. The internal control was *β*-actin (Rabbit IgG, Beyotime, China) at 1 : 1000. An Odyssey CLx Imaging System (LI-COR, USA) was used to detect antibody-bound proteins. Relative protein expression was normalized to *β*-actin and quantified using the ImageJ software.

### 2.6. ROS and Immunofluorescence (IF) Assays

The cells were stimulated with CoCl_2_ for 24 h in a 6-well plate. The level of ROS was detected with a ROS assay kit (Beyotime, China). Images were observed using a fluorescence microscope (Nikon, Japan). The intensity of fluorescence before and after stimulation was measured in real time at 488 nm. For IF, HPDLCs were seeded into cell-climbing slices in a 12-well plate, fixed with 4% paraformaldehyde for 15 min and then treated with 0.5% Triton X-100 for permeabilization. After blocking with 5% bovine serum albumin for 30 min, the cells were incubated overnight at 4°C with primary antibodies (1 : 250) diluted in PBS: TXNIP (Rabbit IgG, Boster, China), NLRP3 (Rabbit IgG, Boster, China), ASC (Rabbit IgG, Santa Cruz, USA), and caspase-1 (Rabbit IgG, Immunoway, China). Finally, the cells were incubated with Alexa Fluor-488 and Alexa Fluor-555 conjugate secondary antibodies (Invitrogen, USA) for 1 h and the nuclei stained with DAPI for 5 min. Images were captured using a fluorescence microscope (Nikon, Japan) and a confocal laser scanning microscope (Nikon, Japan), and the fluorescence intensity was quantified using ImageJ software.

### 2.7. Confocal Microscopy of Inflammasome Proteins in HPDLCs

Cells were handled as in the IF assay and then incubated overnight at 4°C with primary antibodies (1 : 250) diluted in PBS: NLRP3 (Rabbit IgG, Boster, China), ASC (Mouse IgG, Santa Cruz, USA), and caspase-1 (Rabbit IgG, Immunoway, China). Next, the cells were cleaned and labeled with Alexa Fluor-488 and Alexa Fluor-555 conjugate secondary antibodies and then sequentially scanned and visualized using an Olympus laser scanning confocal microscope (Olympus, Japan). Image-Pro Plus software (Media Cybernetics, USA) was used for colocalization analysis, and the Pearson correlation coefficient represented colocalization.

### 2.8. Animals and Unilateral Nasal Obstruction (UNO) Model

A UNO rat model was established by cauterizing the external nostril [24–26]. Briefly, 8-day-old male Wistar rats (*n* = 30) were randomly divided into two groups: SHAM and UNO. Rats in the two groups were first anesthetized by hypothermia (10 min at -20°C), and then, the external nostril of rats in the UNO group was burned using a heated hot container for the gutta-percha point, whereas in the SHAM group, the surrounding tissues of the left nostril were burned. Rats in both groups were raised in the animal facility of Tongji University, Shanghai, China, under specific pathogen-free (Suzhou Fengshi Laboratory Animal Equipment, China) conditions at 22 ± 2°C under a 12 : 12 h light : dark cycle, with food and water provided ad libitum. In the experimental period, body weights were recorded, and nasal obstruction was confirmed every week. Rats whose external nostrils remained blocked throughout the experiment were considered successful models. After 7 weeks, all rats were humanely sacrificed. The periodontal tissue was collected and processed for analysis as describe below. This research protocol was approved by the Ethical Committee of the Tongji University College.

### 2.9. Histological Assessment

Rat periodontal tissue samples were fixed in 4% paraformaldehyde, prepared for paraffin-embedding (Beyotime, China), and sectioned into 4 *μ*m sections. For IF, the sections were incubated overnight at 4°C with primary antibodies (1 : 250) diluted in PBS: TXNIP (Rabbit IgG, Boster, China), NLRP3 (Rabbit IgG, Boster, China), ASC (Rabbit IgG, Immunoway, USA), and caspase-1 (Rabbit IgG, Immunoway, China) and then incubated with Alexa Fluor-488 and Alexa Fluor-555 conjugate secondary antibodies at room temperature for 1 h. For the TUNEL assay, a One-Step TUNEL Assay Kit (Boster, China) was used. All sections were then stained with DAPI (Beyotime, China) for 5 min in the dark. Sections were captured using a fluorescence microscope and a confocal laser scanning microscope, and the fluorescence intensity was quantified using ImageJ software.

### 2.10. Statistical Analysis

All statistical analyses were performed using GraphPad Prism (GraphPad Software Inc., USA). The data are expressed as mean ± SEM. Statistical significance was assessed using one-way ANOVA, and then, the Bonferroni post hoc test was used to test the selected comparison or Dunnett's multiple comparison post hoc test, if each group needed to be compared with the control group. Statistically significant difference was determined at ^∗^*p* < 0.05.

## 3. Results

### 3.1. Systemic Changes and Increased Apoptosis of Periodontal Tissue in UNO Rats

**Figure 1:**
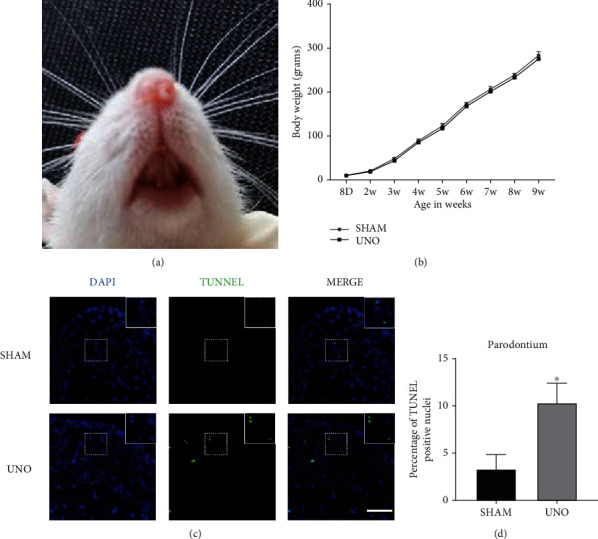
Effects of unilateral nasal obstruction (UNO) in rats. (a) Establishment of UNO in Wistar model rats. (b) Changes in body weight of rats during the experiment (*n* = 5). (c) TUNEL staining of the periodontal ligament. Scale bars = 50 *μ*m. (d) Percentage of TUNNEL-positive nuclei in SHAM and UNO. The above data are presented as the mean ± SEM, ^∗^*p* < 0.05.

The establishment of the UNO rat model was as mentioned above [24–26]. We used the peripheral nostril obstruction in rats as the standard for successful modeling. Body weight was used as an indicator of the influence of hypoxia on rats, and the statistical results showed that there was no significant difference in body weight between the SHAM and UNO groups (Figures 1(a) and 1(b)). TUNEL staining was performed to investigate the effect of hypoxia on periodontal cell apoptosis in UNO rats after UNO for 8 weeks. During apoptosis, the genomic DNA of cells is fragmented; this is detectable by TUNEL staining [27, 28]. Results showed that TUNEL-positive cells were increased in UNO rats compared to those in SHAM rats (Figures 1(c) and 1(d)). Taken together, the results show that apoptosis is a response to oxidative stress and is a product of the inflammatory response.

### 3.2. The Levels of TXNIP/NLRP3 Inflammasome Signaling Pathway-Related Factors in Periodontal Tissues of Rats in the UNO Group Were Changed

**Figure 2:**
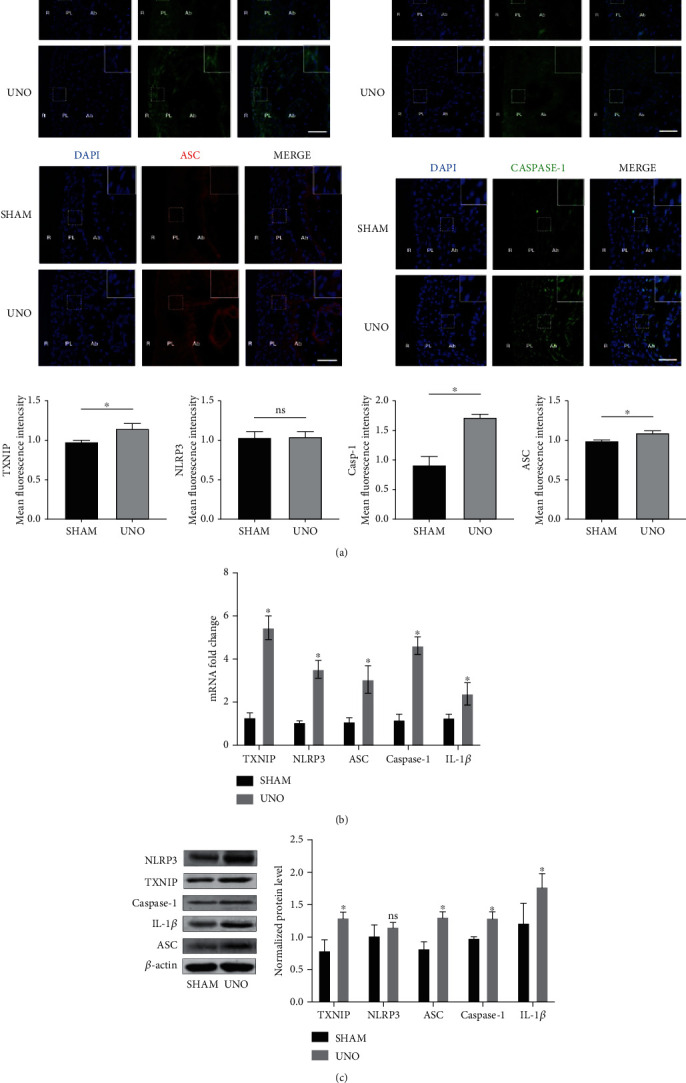
Effects of systemic hypoxia stimulation on the expression of TXNIP/NLRP3 signaling pathway-related factors in periodontal tissues of rats: (a) immunofluorescence staining (IF) showed that TXNIP, NLRP3, ASC, and caspase-1 in periodontal tissues. Ab: alveolar; PL: periodontal ligament; R: root. Scale bars = 25 *μ*m. (b) RT-PCR analysis of TXNIP, NLRP3, ASC, caspase-1, and IL-1*β* in periodontal tissues (*n* = 5). (c) Western blotting analysis of TXNIP, NLRP3, ASC, caspase-1, and IL-1*β* in periodontal tissues (*n* = 5 for each group). The above data are presented as the mean ± SEM, ^∗^*p* < 0.05.

The expression of hypoxia-related factors in periodontal tissues of the UNO and SHAM groups was detected by RT-PCR to study the changes in inflammatory factors in periodontal tissues. The expression of TXNIP, NLRP3, caspase-1, ASC, and the inflammatory factor IL-1*β* was increased in the UNO group compared with the SHAM group. We then detected the protein levels of TXNIP, NLRP3, caspase-1, ASC, and IL-1*β* in the periodontal tissues of the two groups of rats (Figures 2(b) and 2(c)). IF showed that the UNO group had higher TXNIP, ASC, and caspase-1-positive cells than did the SHAM group, whereas there was no difference in NLRP3-positive cells. In addition, western blotting showed higher expression levels of TXNIP, caspase-1, ASC, and IL-1*β* in the UNO group than in the SHAM group, whereas there was no difference in NLRP3 protein expression (Figure 2(a)).

### 3.3. CoCl_2_ Simulates Hypoxia in HPDLCs, Induces ROS Production, Increases TXNIP Expression, and Upregulates NLRP3 Inflammasome-Related Factor Expression

**Figure 3:**
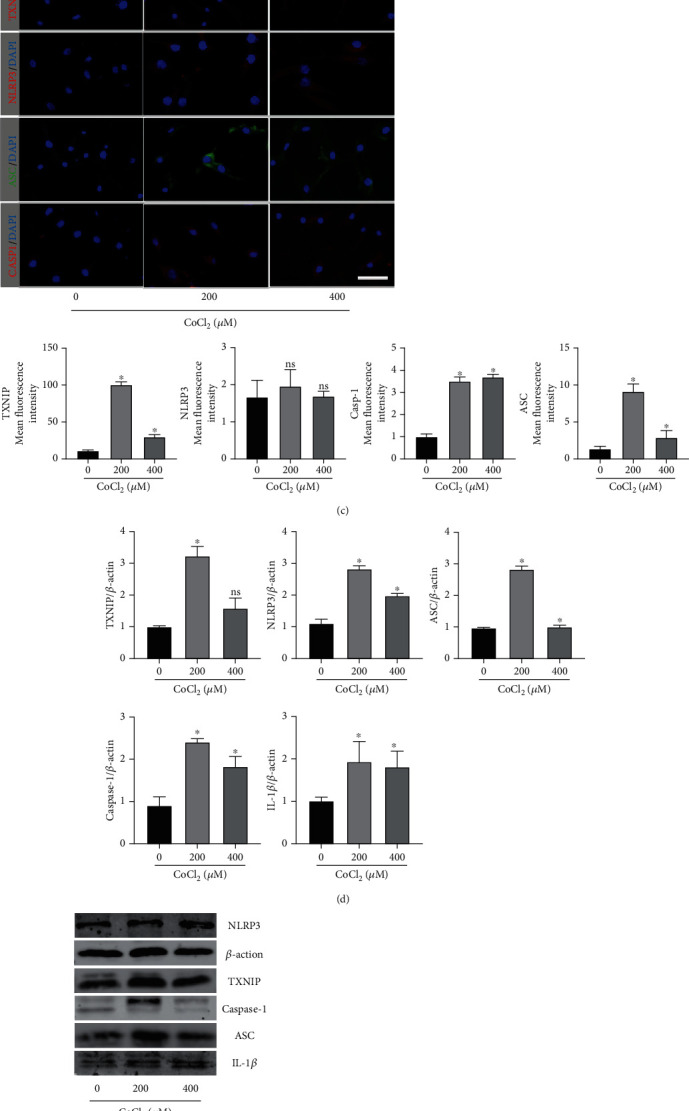
Hypoxic environment induced by CoCl_2_ activates the ROS/TXNIP/NLRP3 inflammasome pathway in periodontal membrane fibroblasts. (a) Effects of different concentrations of CoCl_2_ on the proliferation of periodontal membrane fibroblasts cultured for 24 h and 48 h. (b, c) Immunofluorescence staining (IF) showed that the expressions of ROS, TXNIP, NLRP3, ASC, and caspase-1 were in the cells treated with different concentrations of CoCl_2._. Scale bars = 1 *μ*m. (d) RT-PCR analysis of TXNIP, NLRP3, caspase-1, ASC, and IL-1*β* in HPDLCs cells treated with 200 *μ*M and 400 *μ*M CoCl_2_ for 24 h. (e) Western blotting analysis of TXNIP, NLRP3, caspase-1, ASC, and IL-1*β* in HPDLC cells treated with 200 *μ*M and 400 *μ*M for 24 h. The above data are presented as the mean ± SEM, ^∗^*p* < 0.05.

CoCl_2_-induced hypoxia is one of the most commonly used models in hypoxia studies. To study the effect of hypoxia on HPDLCs, different concentrations of CoCl_2_ were used to stimulate cells. After two days of culture at 200 *μ*M and 400 *μ*M, cell viability decreased significantly. However, there was no significant difference in the cell viability of HPDLCs treated with 200 *μ*M at 24 h (Figures 3(a)). In addition, the fluorescence intensity of ROS, TXNIP, NLRP3, ASC, and caspase-1 differed from those in the normal group (Figures 3(b) and 3(c)). We noted that with a change in CoCl_2_ concentration, NLRP3 expression was not significantly different from that in the control group. At the same time, one day after 200 *μ*M and 400 *μ*M culture, we performed RT-PCR and western blotting analysis on TXNIP, NLRP3, caspase-1, ASC, and IL-1*β* in HPDLCs (Figures 3(d) and 3(e)). The results showed that culture with 200 *μ*M CoCl_2_ for 24 h significantly increased the transcription and expression of TXNIP, NLRP3, caspase-1, ASC, and IL-1*β*. However, with the increase in concentration, the expression of the above factors did not increase, which could result from NLRP3 inflammasome upregulation-regulated gene expression. Therefore, in our subsequent experiments, we used the 200 *μ*M culture for 24 h as a method to construct a hypoxic cell environment.

### 3.4. Inhibition of ROS Production Attenuates CoCL_2_-Induced TNXIP Expression and NLRP3 Inflammasome Activation

**Figure 4:**
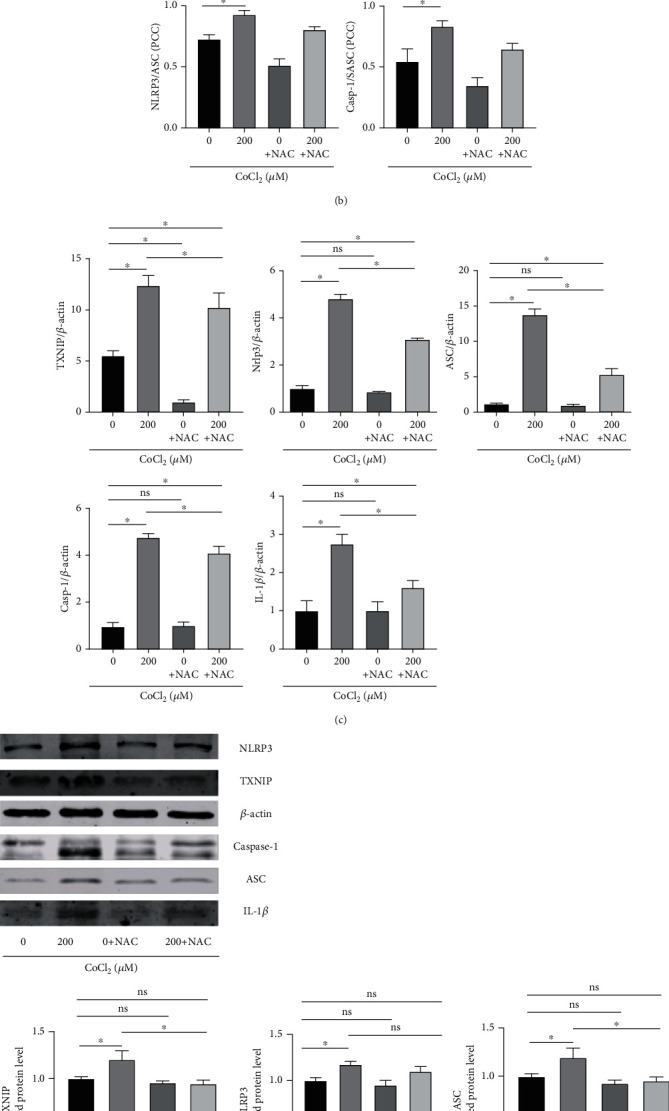
ROS mediates the inflammatory response of periodontal membrane fibroblasts under hypoxia. (a) ROS in HPDLCs after 24 h treatment with or without 2 mM NAC in the normal group and CoCl_2_-induced hypoxia environment. (b) Degree of aggregation of immunofluorescence staining (IF) in periodontal membrane fibroblasts after 24 h treatment with or without 2 mM NAC in the normal group and CoCl_2_-induced hypoxia environment. (c) RT-PCR analysis of TXNIP, NLRP3, ASC, caspase-1, and IL-1*β* in periodontal membrane fibroblasts after 24 h treatment with or without 2 mM NAC in the normal group and CoCl_2_-induced hypoxia environment. (d) Western blotting analysis of TXNIP, NLRP3, ASC, caspase-1, and IL-1*β* in periodontal fibroblasts treated with or without 2 mM NAC for 24 h in the normal group and CoCl_2_-induced hypoxia environment. The above data are presented as the mean ± SEM, ^∗^*p* < 0.05.

NAC inhibited ROS generation (Figure 4(a)). We verified whether ROS are involved in CoCl_2_-induced NLRP3 inflammasome activation and expression by NAC pretreatment (Figure 4(b)). The results showed that the CoCl_2_-induced aggregation of NLRP3 inflammasome-related factors and IL-1*β* expression decreased significantly with decreased ROS expression. Interestingly, NAC also inhibited CoCl_2_-induced TXNIP expression (Figures 4(c) and 4(d)).

## 4. Discussion

OSAHS causes a decrease in blood oxygen saturation. With a decrease in blood saturation, OSHAH patients often develop cardiovascular system and cognitive disorders. Recent literature indicates that the incidence of periodontal disease is higher in patients with OSHAS than in healthy individuals. However, the mechanism underlying the correlation between OSAHS and the incidence of periodontal disease remains unclear. It is important to determine how hypoxia affects the biological behavior of HPDLCs, the principal cells in the periodontal ligament that help maintain the stability of the periodontal system and promote the repair and regeneration of periodontal tissue [29].

Hypoxia can lead to oxidative damage and apoptosis in various tissues, which is key to maintaining tissue homeostasis [30–32]. Many factors can trigger its occurrence, especially oxidative stress [33]. Elevated ROS levels and oxidative stress were observed in patients with OSAHS. In vivo, we observed more positive TUNEL signals in the periodontal membrane of rats with UNO. These results indicate that hypoxia can lead to oxidative stress and apoptosis in periodontal tissue. After modeling UNO in rats, rats presented with oral respiratory symptoms [24–26]. Oral respiration is one of the main clinical manifestations in patients with OSAHS [1]. Oral respiration is also reported to be associated with gingival inflammation [34, 35]. The NLRP3 inflammasome is a multiprotein complex composed of NLRP3, ASC, and caspase-1. When the NLRP3 inflammasome polymerizes, IL-1*β* is produced to induce a cellular inflammatory response. Previous studies have shown that hypoxia is involved in NLRP3 activation and subsequent inflammasome formation by increasing TXNIP expression [36–38], and the occurrence of periodontitis is associated with the NRLP3 inflammasome [39]. Recent studies have reported that TXNIP could induce the formation and aggregation of the NLRP3 inflammatory body, leading to dysfunction and injury of periodontal membrane fibroblasts, resulting in periodontitis [10]. The in vivo results showed that the transcription and expression of TXNIP, ASC, and caspase-1 in periodontal tissues of UNO rats were higher than those of SHAM rats, and the expression level of the inflammatory factor IL-1*β* in periodontal tissues of UNO rats was upregulated. In IF experiments, high TXNIP, ASC, and caspase-1 expression signals were detected in the periodontal membrane of UNO rats compared to those of SHAM rats. Interestingly, our experimental results showed that compared to that in the SHAM group, the transcription level of NLRP3 in periodontal tissues was increased in the UNO group; however, there was no significant difference in its protein level. The result that the NRLP3 transcription level in periodontal tissues was upregulated but the protein level was not upregulated in a hypoxic environment in vivo was consistent with the results of a study, which demonstrated that hypoxia mainly leads to the aggregation of inflammasomes by increasing the nuclear localization of NLRP3 and caspase-1 [40], resulting in increased expression of inflammatory factors in tissues. Meanwhile, it is reported that there are no differences in the serum levels of NLRP3 inflammasome components between OSAHS and controls [41]. Hypoxia-induced cell death may play a key role in systemic diseases caused by OSAHS. In addition, the expression level of the inflammatory factor IL-1*β* in the periodontal tissues of UNO rats was upregulated.

In vitro experiments found that CoCl_2_ stimulation of HPDLCs resulted in ROS production and accumulation in cells, but the accumulation of ROS in cells was significantly improved after the application of NAC. Meanwhile, NAC significantly reduced the level of the inflammatory gene IL-1*β* and the aggregation of NLRP3 inflammasome. Several studies have shown that targeting ROS can reduce cardiovascular damage in OSAHS [42]. Therefore, antioxidant therapy may be a new treatment method to reduce the damage of periodontal tissue caused by oxidative stress.

OSAHS is difficult to diagnose, and patients themselves tend to ignore and underestimate the symptoms [43]. Therefore, it is necessary to introduce specific sleep disorder questions and questionnaires into dental records, which can help clinicians identify patients at risk for OSAHS [44]. The study suggests that there is a pathological correlation between OASHS and the occurrence of periodontal diseases, which is supported by the above views.

## 5. Conclusions

In summary, our study suggests that OSAHS may increase the expression of inflammatory factors in periodontal tissues through the ROS/TXNIP/NLRP3 inflammasome signaling pathway, thus making periodontal tissues more prone to inflammatory lesions.

## 6. Limitation

The animal model of this experiment lacks anatomical factors of upper airway stenosis, making it significantly different from human OSAHS. In future experiments, we will continue to study how to establish animal models that are closer to the pathogenesis of human OSAHS. The effect of UNO modeling on periodontitis was not determined in this study. This part of the experiment will be supplemented in future studies to explore whether OSASH can aggravate the severity of periodontitis.

## Figures and Tables

**Table 1:** 

Gene	Forward (5′–3′)	Reverse (5′–3′)
*β*-Actin (human)	CTCGCCTTTGCCGATCC	TCTCCATGTCGTCCCAGTTG
NLRP3 (human)	CTGGCATCTGGGGAAACCT	TTAGGCTTCGGTCCACACAG
TXNIP (human)	TCAGTATTGCAGGGCTTGGC	GTCTCTTGAGTTGGCTGGCT
ASC (human)	ATCCAGGCCCCTCCTCAG	AGAGCTTCCGCATCTTGCTT
Caspase-1 (human)	ACAAGACCTCTGACAGCACG	TTCACTTCCTGCCCACAGAGAC
IL-1*β* (human)	TTCGACACATGGGATAACGAGG	TTTTTGCTGTGAGTCCCGGAG
TNF-*α* (human)	CTGGGCAGGTCTACTTTGGG	CTGGAGGCCCCAGTTTGAAT
*β*-Actin (human)	GGCACAGTCAAGGCTGAGAAT	ATGGTGGTGAAGACGCCAGTA
NLRP3 (rat)	CTGCAGAGCCTACAGTTGGG	GTCCTGCTTCCACACCTACC
TXNIP (rat)	TCCACAGATGGGTGGCAATC	AAGTGGGCCAGGTCTGAATG
ASC (rat)	GCACAGCCAGAACAGAACATT	CCAGGCTGGAGCAAAGCTAA
Caspase-1 (rat)	GACCGAGTGGTTCCCTCAAG	GACCGAGTGGTTCCCTCAAG
IL-1*β* (rat)	GACCTGTTCTTTGAGGCTGA	TCCATCTTCTTCTTTGGGTATTGT

## Data Availability

The datasets used and/or analyzed during the current study are available from the corresponding author upon request.
